# The Effect of Statins on Markers of Breast Cancer Proliferation and Apoptosis in Women with In Situ or Early-Stage Invasive Breast Cancer

**DOI:** 10.3390/ijms25179587

**Published:** 2024-09-04

**Authors:** Anam Kamal, Julie Boerner, Hadeel Assad, Wei Chen, Michael S. Simon

**Affiliations:** 1 Ascension Providence Hospital, Michigan State University, Southfield, MI 48075, USA; 2 Karmanos Cancer Institute, Wayne State University, Detroit, MI 48201, USA; boernerj@karmanos.org (J.B.); assadh@karmanos.org (H.A.); chenw@karmanos.org (W.C.)

**Keywords:** breast cancer, statins, window-of-opportunity trial, Ki-67, cyclin D-1, CC3, P27

## Abstract

Statins, inhibitors of HMG-CoA reductase, have been shown to have potential anti-carcinogenic effects through the inhibition of the mevalonate pathway and their impact on Ras and RhoGTAases. Prior studies have demonstrated a reduction in breast tumor proliferation, as well as increased apoptosis, among women with early-stage breast cancer who received statins between the time of diagnosis and the time of surgery. The aim of this study was to evaluate the impact of short-term oral high-potency statin therapy on the expression of markers of breast tumor proliferation, apoptosis, and cell cycle arrest in a window-of-opportunity trial. This single-arm study enrolled 24 women with stage 0-II invasive breast cancer who were administered daily simvastatin (20 mg) for 2–4 weeks between diagnosis and surgical resection. Pre- and post-treatment tumor samples were analyzed for fold changes in Ki-67, cyclin D1, p27, and cleaved caspase-3 (CC3) expression. Out of 24 enrolled participants, 18 received statin treatment and 17 were evaluable for changes in marker expression. There was no significant change in Ki-67 expression (fold change = 1.4, *p* = 0.597). There were, however, significant increases in the expression of cyclin D1 (fold change = 2.8, *p* = 0.0003), p27 cytoplasmic (fold change = 3.2, *p* = 0.025), and CC3 (fold change = 2.1, *p* = 0.016). Statin treatment was well tolerated, with two reported grade-1 adverse events. These results align with previous window-of-opportunity studies suggesting a pro-apoptotic role of statins in breast cancer. The increased expression of markers of cell cycle arrest and apoptosis seen in this window-of-opportunity study supports further investigation into the anti-cancer properties of statins in larger-scale clinical trials.

## 1. Introduction

Statins are competitive inhibitors of Hydroxy Methyl Glutaryl Co-enzyme A (HMG-CoA) reductase, the rate-limiting enzyme in the cholesterol biosynthesis pathway, and have a known favorable impact on cardiovascular mortality [1]. Statins also have possible anti-carcinogenic effects through the inhibition of the mevalonate pathway and downstream impact on Ras and RhoGTAases [2,3,4,5,6]. Notably, cellular uptake of lipophilic statins may be related to their inhibition of cell growth [7,8,9,10,11]. Despite the biological rationale suggesting a protective impact on cancer risk, population-based studies of statins have yielded mixed results, with some studies showing a protective effect [12,13,14,15] and others suggesting increased risk or no association [16,17,18,19]. Two meta-analyses of the relationship between statins and breast cancer risk showed no significant association [20,21]. Other studies demonstrated a relationship between statins and earlier-stage breast cancer at diagnosis [22,23], lower relapse rates [24,25,26], and decreased cancer mortality [23,27,28].

The rationale for the anti-cancer effect of statins has been shown in preclinical studies with in vitro–cell line data showing anti-proliferative, apoptotic, and anti-invasive properties [3,29,30,31,32,33]. As inhibitors of the mevalonate pathway, statins exhibit complex anti-tumoral mechanisms that encompass both cholesterol-dependent and cholesterol-independent effects. The cholesterol-dependent effects involve depriving tumor cells of the cholesterol they require for increased uptake [34,35]. Conversely, the cholesterol-independent pleiotropic effects of statins may also play a role in their anti-tumoral properties [36,37].

By inhibiting HMG-CoA reductase, statins reduce the levels of mevalonate and lipid isoprenoid intermediates such as farnesyl pyrophosphate (FPP) and geranylgeranyl pyrophosphate (GGPP) [38]. These intermediates provide lipid attachments for intracellular G-proteins like Ras and Rho, which need to undergo post-translational prenylation by FPP or GGPP to move from the cytoplasm to the cell membrane [38]. By inhibiting the prenylation of Ras and Rho proteins, statins may suppress various downstream signaling pathways, such as the PI3K/Akt/mTOR and MAPK/ERK pathways, which are often disrupted in many cancer types. A phase II window-of-opportunity study that enrolled women with early-stage breast cancer indicated that atorvastatin might suppress the MAPK pathway [39]. Other studies of breast and lung cancer cell lines demonstrated statin-induced decreases in expression of anti-apoptotic BCL-2 and increases in pro- apoptotic BAX protein [40]. In a prior window-of-opportunity study of women with stage 0 and 1 breast cancer, administration of pre-surgical fluvastatin was associated with a reduction in breast tumor proliferation, as demonstrated by a reduction in Ki-67 expression in high-grade tumors and a preferential increase in tumor apoptosis in high-grade and ER-negative tumors [41]. A second window-of-opportunity study demonstrated the greatest reduction in Ki-67 expression associated with statin use in breast tumor samples which had high expression of HMGCoAR [42]. Other in vitro analyses suggest that statin antiproliferative action may be due to an effect on cyclin D1 (oncogene) and p27 (tumor suppressor) through up-regulated expression of p27 and down-regulated expression of cyclin D1 in breast cancer cells [43].

A more recent meta-analysis provided some evidence that patients with non-metastatic triple-negative breast cancer (TNBC) who received statins concurrently with oncologic treatment (surgery, chemotherapy, radiation) showed improvements in long-term DFS. The analysis showed a significant increase in 5-year DFS (OR 1.44, 95% CI 1.04–1.98, *p* = 0.03), but no improvement in OS [44]. A different study examined the effects of both simvastatin and atorvastatin, finding that their inhibitory effects were significantly stronger in TNBC cell lines compared to non-TNBC cell lines [45]. Additionally, the study assessed the impact of combining simvastatin with two commonly used TNBC treatments, docetaxel and doxorubicin. This combination of cytotoxic drugs and simvastatin synergistically enhanced growth inhibition in the two TNBC cell lines that were tested. Moreover, the study determined that cell lines with mutant p53 responded more effectively to both statins than those with wild-type p53, indicating that p53 mutational status could be a predictive biomarker for statin response.

The goal of the current analysis is to replicate the results of the prior two window-of-opportunity studies [41,43] and to evaluate the impact of a short-term oral, high-potency statin on changes in expression of predictive markers of breast tumor proliferation (Ki-67 and cyclin D1) and markers of apoptosis (CC3) and cell cycle arrest (p27).

## 2. Results

**Figure 1:**
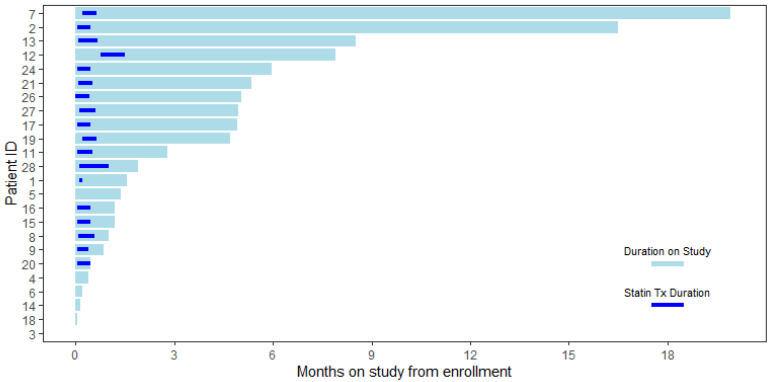
Swimmer plot for treatment and duration of the study.

Table 1 shows a comparison of all participants stratified by those that were evaluable and non-evaluable in regards to demographics and disease characteristics. The median age was 61 (range 42–73), 79% were White and 21% Black, the majority had stage I or 2 disease (70%), and the majority were ER-positive (88%) and HER2neu negative (71%). All of the participants were women, and only those with stage I–II disease were checked for HER2neu status. The ER/PR/HER2neu tumor phenotype of the 11 evaluable women with stage I disease is shown in the supplementary data (Table S1). There were no significant differences in demographic or clinical characteristics between the evaluable and non-evaluable cohorts, except that evaluable women were more likely to be diagnosed at stage 0 (DCIS) and none of the evaluable women had stage II disease. For the 17 evaluable women, statin treatment occurred for a median of 14 days (Range 12–27). The period of treatment and total duration of the study for each participant are shown in Figure 1.

**Figure 2:**
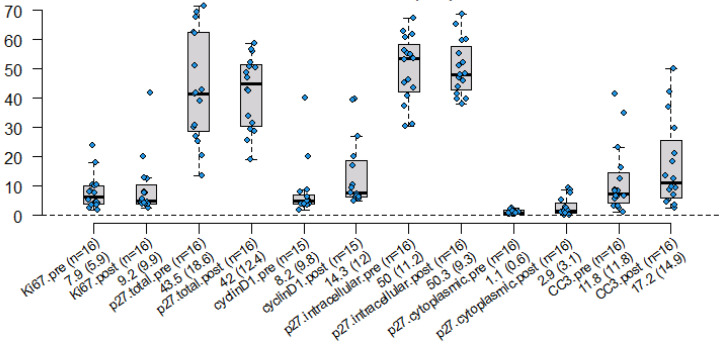
Distribution of molecular markers of cell proliferation, apoptosis, and cell cycle arrest pre- and post-Statin Treatment with Mean (std).

To determine if statin treatment resulted in a decrease in cellular proliferation, increased apoptosis, or cell cycle arrest, paired tumor samples were immunohistochemically stained for the proliferative and cell cycle markers Ki67 and cyclin D1, the cell cycle arrest marker p27, and the apoptosis marker cleaved caspase 3 (CC3). Figure 2 shows the distributions of biomarkers before and after completion of statin treatment. There was no apparent difference in the markers of proliferation as determined by the percentage of positive cells (Ki67); however, there was a fold change in cyclin D1 (the other marker of proliferation), as well as in the cell cycle arrest marker P27 (cytoplasmic) and the marker of apoptosis CC3.

**Figure 3:**
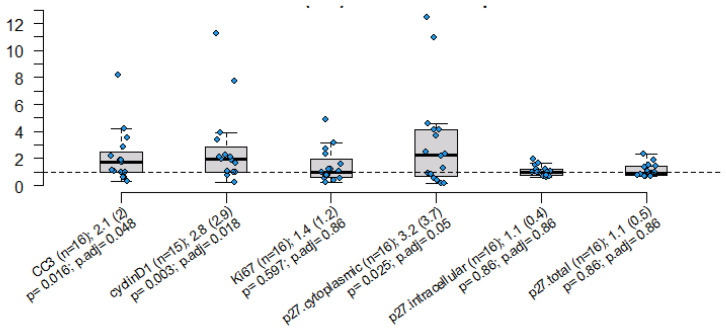
Distribution of fold change between pre- and post-treatment markers with mean (std) and Wilcoxon *p*-values. Adverse events were reported. One patient developed a musculoskeletal and connective tissue disorder, which was attributed to simvastatin. The other patient developed a bladder infection, which was unrelated to the simvastatin.

Figure 3 shows the distribution of the “fold” change in the pre- and post-treatment values of the markers of proliferation, cell cycle arrest, and apoptosis. The measured “fold change” for Ki-67 was 1.4, with a *p*-value of 0.597 and an adjusted *p*-value of 0.86, and the fold change for cyclin D-1 was 2.8, with a *p*-value of 0.0003 and an adjusted *p*-value of 0.018. The fold changes for p27 cytoplasmic and CC3 were 3.2, with a *p*-value of 0.025 and an adjusted *p*-value of 0.05, and 2.1, with a *p*-value of 0.016 and an adjusted *p*-value of 0.048, respectively. There were no significant differences in fold change for P27, either total or intracellular.

## 3. Discussion

The chemical properties of statins, such as lipophilicity, affect their cellular uptake and subsequent anti-tumor effects. Lipophilic statins have better access to various tissues, including cancer cells [46]. These statins are taken up by cells through the organic anion-transporting polypeptide OATP1B1, which is mainly expressed by hepatocytes, and lipophilic statins can also enter cells through passive diffusion across the membrane. Consequently, hydrophilic statins have a higher affinity for hepatic tissue, but do not readily accumulate in other tissues. In contrast, lipophilic statins reach higher levels in extrahepatic tissues, where they can interfere with cholesterol synthesis [47,48,49]. Multiple in vitro studies on different cancer cell lines have shown that lipophilic statins have a superior anti-tumor effect compared to hydrophilic statins [46].

A study by Beckwitt et al. [50] utilized four cancer cell lines from different primary tumors and compared the anti-tumor activity of four statins—atorvastatin, simvastatin, rosuvastatin, and pravastatin. The cancer cell lines included those from breast (MCF-7 and MDA-MB-231), prostate (DU145), brain (SF-295), and melanoma tumors. Atorvastatin demonstrated the highest anti-tumor activity, whereas pravastatin was the least effective at inhibiting tumor growth in all the cell lines studied. Simvastatin and atorvastatin were equally effective, but rosuvastatin was less potent than atorvastatin [50]. Jiang et al. had previously reported similar superior outcomes for lipophilic statins against breast cancer (MDA-MB-231, MDA-MB-432, MDA-MB-435) and brain cancer (A172, LN443, U87, U118, U251) cell lines compared to rosuvastatin and pravastatin, which are both hydrophilic [51].

This window-of-opportunity study utilized simvastatin, a lipophilic statin, based on previous reports of improved potency and an anti-tumor effect, as well as easy availability. The study evaluated its impact on breast cancer proliferation, cell cycle arrest, and apoptosis and found no statistically significant differences in Ki-67, a marker of cell-proliferation, pre- or post- treatment, which was the primary endpoint of our study. There was, however, an increase in cyclin D-1 post-treatment (another marker of cellular proliferation), as well as an increase in the cell cycle arrest and apoptosis markers p27 cytoplasmic and CC3, respectively.

Ki-67 is expressed in all active phases of the cell cycle (G1, S, G2, M), but is absent in resting cells (G0), and has been established as a prognostic tool predicting relapse-free and overall survival in breast cancer patients [52,53,54]. Other studies evaluating the impact of statins on biological parameters are summarized in Table 2. In a study comparing doses of the lipophilic statin Fluvastatin (20 mg or 80 mg), Garwood et al. demonstrated a significant reduction in Ki-67 expression only among women with “high-grade” and ER-negative tumors. A total of 40 out of 45 patients who enrolled completed the protocol; 29 had paired Ki-67 primary endpoint data. Proliferation of high-grade tumors decreased by a median of 7.2% (*p* = 0.008), which was statistically greater than the 0.3% decrease in proliferation for low-grade tumors. In this analysis, the fluvastatin dose had no impact on the change in Ki-67 levels [41]. In comparing these results to our data, 35% of the women (N = 6/17) who were evaluable for biomarker changes in our study had DCIS and were therefore not evaluable for tumor grade. We did not collect information regarding tumor grade for the remaining eleven women who had stage 1 breast cancer, and therefore, it is possible that the lack of variability and the absence of high-grade tumors in our small cohort is the reason why we did not observe a decrease in Ki-67 after statin treatment. Additionally, only two women had ER-negative tumors in our analysis, which could also account for the lack of change in Ki-67, as seen in the Garwood study.

Likewise, in a 2nd window-of-opportunity study of atorvastatin (80 mg), there was a significant decrease in Ki-67 expression post-statin use only among women that expressed HMGCoAR in their tumors [42]. Similar findings were seen in a smaller study [45], although those results were not statistically significant, as well as in a study which used Cyclin D-1 as a marker of proliferation [43].

Our results also demonstrated a significant fold increase in cyclin D1 pre- and post-statin use, another marker of cell proliferation, and a regulator of G1/S transition through its interaction with CDK4 and CDK6. This interaction led to inactivation of the Rb-protein and expression of proliferation-associated target genes [55,56]. Interestingly, the fold change seen in our study involved an increase in the expression of cyclin D1 post-statins, which contrasts with the other report cited above, which suggested an anti-proliferative action of statins through the downregulation of the oncogene cyclin D1 [43]. While our results cannot explain this discrepancy, it is worth noting that the Feldt study had a larger number of participants and used a high-intensity dose of atorvastatin at 80 mg/day, in contrast to the lower dose of simvastatin used in our trial. In the Garwood study, the dose of fluvastatin (80 vs. 20 mg) did not impact the results, although the time period of statin use in the Garwood trial (3–6 weeks) was somewhat longer than we achieved in our trial (median 14 days) (Table 2).

In addition, our analysis also showed a significant 3.2-fold change in the level of p27 cytoplasmic pre- and post-statins, which is a key biomarker of cell cycle arrest. This specific biomarker not only exhibited a noteworthy increase after short-term statin treatment, but this change also achieved statistical significance with an FDR-adjusted *p*-value. This finding supports the notion that statins may play a role in promoting cell cycle arrest in breast cancer cells and aligns with findings from the Feldt study (Table 2) [43].

Caspases are a family of cysteine-dependent, aspartate-specific proteases which also play a pivotal role in initiating and executing apoptosis. Among the family of proteases, caspase-3 emerges as a specific effector and transmits an apoptotic signal by enzymatically acting on downstream targets, including poly ADP ribosome polymerase (PARP) and other substrates [57]. Our results showed a significant fold increase in caspase-3 pre- and post-statins, which is another finding of potential importance. Our results substantiate the results of Garwood et al., which demonstrated that paired data for CC3 showed that tumor apoptosis increased in 38%, remained stable in 41%, and decreased in 21% of patient samples [41].

**Table 2:** Findings from window-of-opportunity studies utilizing statins in early-stage breast cancer.

Study/Reference	Type of Statin and Dose	Duration of Statin Treatment Prior to Surgery	Study Population	Marker of Proliferation	Marker of Apoptosis/Cell Cycle Arrest	Comments
Garwood et al., 2010 [41]	Fluvastatin 20 mg vs. 80 mg	3–6 weeks	N = 40 DCIS, high and low-grade early-stage IDC (29 women had paired Ki-67 data)	Ki-67 7.2% decrease in high grade tumors	CC3 38% increase (60 vs. 13% in high vs. low-grade tumors)	Significant reduction in Ki-67 expression only among women with “high-grade”, ER-negative tumors No differences in Ki-67 level in the Garwood study based on fluvastatin dose
Bjarnadottir et al., 2013 [42]	Atorvastatin 80 mg	2 weeks	N = 42 Early-stage IDC (26 women had paired Ki-67 data)	Ki-67 Decreased post-treatment (24% vs. 21.9%)	-	Significant reduction in Ki-67 expression only among women with tumors with baseline * HMGCoAR expression
Wang et al., 2015 [58]	Simvastatin 20 mg	5–38 days	N = 15 Early-stage IDC	Ki-67 Decreased post vs. pre-treatment (57.7 ± 35.2 vs. 74.6 ± 59.9)	CC3 Increased post- vs. pre-treatment (23.4 ± 24.3 vs. 8.9 ± 7.4	Change in Ki-67 not statistically significant
Feldt et al., 2015 [43]	Atorvastatin 80 mg	2 weeks	N = 42 Early-stage IDC (30 and 33 women had paired cyclin D-1 and p27 data respectively)	Cyclin D-1 (cytoplasmic intensity) Decreased in 14/30, unchanged 13/30 and increased 3/30 post treatment	P27 (cytoplasmic intensity) Increased in 12/33, unchanged 18/33, decreased 3/33	-

* HMGCoAR: HMG-CoA reductase.

A major limitation of our trial included low participant accrual, which led to a small sample size. One possible reason for this is that our center is a comprehensive cancer center and functions mostly as a referral site for women with advanced breast cancer, suggesting that the availability of eligible women with early-stage breast cancer or DCIS was somewhat limited. Furthermore, women that presented to our center were approached for enrollment after their initial visit with a breast surgeon following a definitive breast biopsy, and this period in their breast cancer diagnosis may have influenced their readiness to delay definitive treatment in order to participate. Also, given the fact that statins are commonly prescribed for the prevention of coronary artery disease, many potential participants were already taking statins and therefore were not eligible. Additionally, it is likely that the COVID-19 pandemic impacted patient enrollment and the timing of surgery due to the priority given to emergent procedures. The median duration of statin treatment in this study was only two weeks, after which only one set of post-treatment tumor markers was obtained from the surgical specimen. The short follow-up period and the absence of surveillance data and information on tumor recurrence are other limitations of our study. Furthermore, our study did not collect information regarding tumor grade. Given the fact that there were only two participants that had ER-negative tumors, and none of the evaluable participants were HER2neu-positive, we did not have the power to determine whether statins had a differential impact based on hormone receptor or HER2neu status. This may limit the generalizability of our results, as we cannot confirm whether statins had a preferential impact on women with high-grade and/or triple-negative tumors, as observed in other studies.

Despite these limitations, we believe that our results add to the literature suggesting a potential pro-apoptotic role and cell-arrest property of statins. Although various clinical and in vitro studies have demonstrated the anti-cancer effects of statins in breast cancer, particularly in the TNBC subtype, these drugs should not be used as standalone treatments for this type of breast cancer. Two prospective clinical trials investigating the role of statins in the neoadjuvant treatment of TNBC have completed enrollment and may provide evidence to either support or refute the use of statins in this context (ClinicalTrials.gov identifier: NCT03358017 and NCT03872388).

## 4. Material and Methods

### 4.1. Trial Design

The trial was designed as a phase II “window of opportunity” study in which the treatment-free window between breast cancer diagnosis and surgical resection was used to study the biological effect of simvastatin, a lipophilic, high-potency statin. As a non-randomized trial, all patients received 20 mg of daily simvastatin for at least two weeks and no longer than four weeks in the interval between their diagnosis and definitive surgery. This was a single-institution trial conduced at the Karmanos Cancer Center (KCC) in Detroit, MI, USA, with repeated biomarker measurements prior to statin treatment and after completion of statins but prior to surgery using pathological specimens. The study was conducted in accordance with the Declaration of Helsinki and the International Conference on Harmonization Good Clinical Practice guidelines. The study was registered at ClinicalTrials.gov (ID number NCT03454529); however, it did not meet the accrual goal of 50 patients and, therefore, did not reach completion. All patients provided written informed consent.

### 4.2. Participants

**Figure 4:**
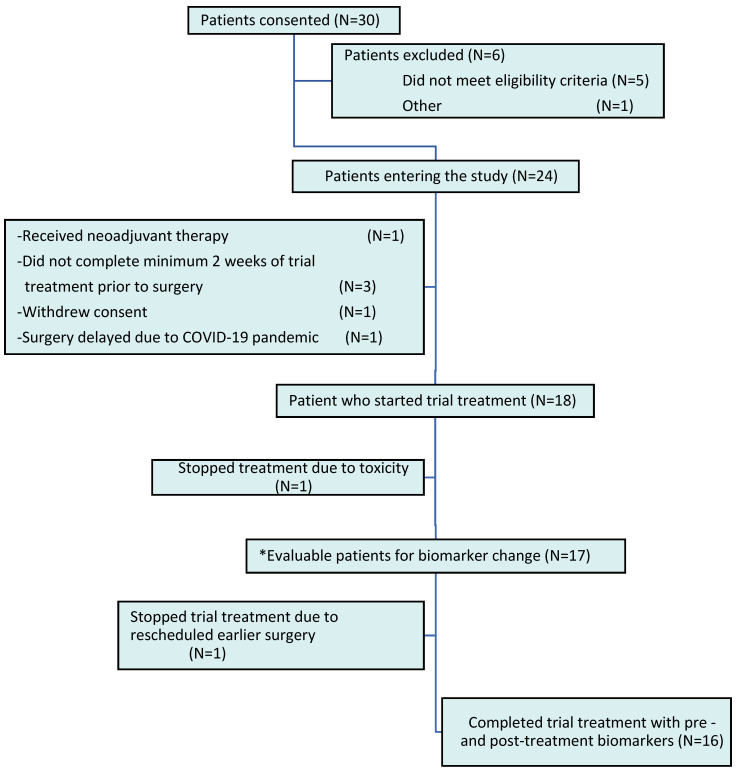
Consort diagram. Participants were enrolled from March 2018 to April 2021. * Evaluable patients were defined as those who received >80% of the trial treatment dose.

Non-pregnant women with clinical stage 0 (in situ) or stage I or II invasive breast cancer were enrolled during the period of time between definitive breast cancer diagnosis by core needle biopsy and final surgical removal of the tumor. A European Cooperative Oncology Group (ECOG) performance status of 0–2 was required, but any measure of ER, PR, or HER2neu expression was permitted. Exclusion criteria included administration of neoadjuvant chemotherapy or hormonal therapy, use of statins or fibrates within three months of enrollment, proven hypersensitivity to statins, and current pregnancy or lactation. Women with newly diagnosed breast cancer were scheduled for surgical resection within four weeks of their initial diagnosis. The study began enrolment in March 2018, and by August 2021, 30 patients had consented, out of which 25 were eligible and 24 went onto the study (Figure 4).

Of the 30 women who consented, only 24 enrolled; 5 did not meet the eligibility criteria and 1 other withdrew consent. Of the 24 women, 1 turned out to be ineligible because she had neoadjuvant hormonal therapy, 3 others did not complete at least two weeks of therapy, 1 withdrew consent, and 1 had a delay in her surgery due to the COVID-19 pandemic, leaving 18 who received statin treatment. Finally, 17 participants were evaluable for changes in marker expression, as 1 participant discontinued statins after three days due to reported side effects. However, only 16 women had both pre- and post-marker measurements, given that one woman stopped statin treatment due to a rescheduled early surgery.

### 4.3. Study Procedures, Endpoints, and Biomarker Evaluation

Eligible and consenting patients were enrolled at the time of the first visit with a breast surgeon after their definitive breast biopsy. After registration, extra biopsy tissue blocks from the initial biopsy specimen were requested for immune-histochemical (IHC) staining. Simvastatin was obtained from commercial sources and provided free of charge to the participant through the KCC pharmacy. Participants were instructed to take simvastatin 20 mg daily for at least two weeks and no longer than four weeks, from the time of consent to the day prior to their breast surgery. A pill diary was maintained through the clinical trials office, which showed that all evaluable patients, except two, completed their full doses during the treatment duration. One patient missed one dose, and another missed two doses. At the time of the lumpectomy or mastectomy, two additional portions of tumor, not required for pathological diagnosis, were formalin-fixed and paraffin-embedded. Specimens were processed by the Pathology Department at the originating hospital based on their CAP/CLIA-based SOPs. If insufficient tissue was available, the patients were deemed ineligible for the study.

Pre- and post-treatment tumor specimens were analyzed for molecular markers of breast tumor proliferation, apoptosis, and cell cycle arrest. These specimens were collected at study entry and after the final surgery and stored for future analysis. Unstained slides were utilized from biopsy and lumpectomy/mastectomy specimens with permission from the Pathology Department at the originating hospital. Formalin-fixed paraffin-embedded (FFPE) sections were cleared through a series of xylenes and alcohol rinses and re-hydrated with tap water. Endogenous peroxidases were blocked with a methanol and 3% hydrogen peroxide solution for twenty minutes. Heat-induced epitope retrieval (HIER) was performed with a decloaker and a pH 9 or pH 6 retrieval buffer for twenty minutes at 95 °C, followed by a pre-protein block for thirty minutes.

The antibodies, suppliers, and dilutions used for IHC staining included: Ki67 1:75 (Dako/Agilent M7240, Santa Clara, CA, USA), p27 1:75 (Dako/Agilent M7203), Cyclin D1 1:100 (Dako/Agilent M3642), and Cleaved Caspase3 1:1000 (Cell Signaling 9664, Danvers, MA, USA). Sections were incubated with the primary antibodies at 4 °C overnight. The staining was developed using Origen Polink-2 Plus HRP Broad Spectrum DAB Detection Kit (Origene, Rockville, MD, USA) and used according to the kit instructions (D41-110), then counterstained with hematoxylin. The IHC slides were analyzed using a Leica Aperio CS2 scanner (Leica, Deer Park, IL, USA) and Halo quantification and imaging software from Indica Labs (Alburquerque, NM, USA); therefore, no pathologists were involved in the reading of these images. A negative and a positive control were identified for each antibody and used to set the parameters for positive staining using Halo, version 3. The areas of tissue containing tumor cells were identified, and the percent of positive cells was quantified for Cyclin D1, Ki-67, and p27. Further, p27 was classified as cytoplasmic, and the intensity of the staining was measured in optical density.

The primary endpoint was the pre- and post-treatment change in Ki-67 expression, a marker of breast tumor proliferation, expressed as a fold change in the percentage of Ki-67 positive cells in the tumor. The secondary endpoints included a change in cyclin D1 (another marker of breast tumor proliferation), also analyzed as a fold change in the percentage of cyclin D1-positive cells. Other secondary end points included P27 (a marker of cell cycle arrest), including the percentage change of total and intracellular P27, as well as cytoplasmic P27, measured as “cytoplasmic intensity”. Cytoplasmic P27 served as an additional biomarker of cell cycle arrest, which was also evaluated in the Feldt study [38]. Lastly, caspase-3 (CC3), a marker of apoptosis, was measured as a percentage of positive cells.

### 4.4. Statistical Analysis

Evaluable participants for efficacy (per-protocol population) were defined as those who received statin treatment with >80% of the scheduled doses. All women who received at least one dose of statins were included in safety analyses.

Descriptive statistics were provided with 95% confidence intervals. The Wilcoxon signed-rank test was used for preliminary efficacy endpoints evaluating fold change and the ratio between post- and pre-treatment measurements. As the number of non-evaluable patients was unexpectedly high, the post hoc Wilcoxon rank sum test for continuous variables or Fisher’s exact test for categorical variables was performed to compare the patients’ baseline characteristics between the evaluable and non-evaluable groups. All correlative studies were descriptive and exploratory. False discovery rate (FDR)-adjusted *p*-values were reported along with the raw p values due to multiple comparisons. Statistical analyses were performed with R version 4.1.0.

## Figures and Tables

**Table 1:** Baseline comparison of evaluable and non-evaluable participant demographics and disease characteristics.

Variable	All (N = 24)	Non-Evaluable (N = 7)	Evaluable (N = 17)	*p*-Value
**AGE**				0.075
Median (range)	61 (42, 73)	55 (42, 71)	63 (48, 73)	
**RACE**				0.126
Black or African American	5 (21%)	3 (43%)	2 (12%)	
White	19 (79%)	4 (57%)	15 (88%)	
**STAGE**				0.03
DCIS (0)	7 (29%)	1 (14%)	6 (35%)	
I	14 (58%)	3 (43%)	11 (65%)	
II	3 (12%)	3 (43%)	0 (0%)	
**ER**				1
Negative	3 (12%)	1 (14%)	2 (12%)	
Positive	21 (88%)	6 (86%)	15 (88%)	
**PR**				0.625
Negative	7 (29%)	1 (14%)	6 (35%)	
Positive	17 (71%)	6 (86%)	11 (65%)	
**^⊥^ HER2**				0.38
** Negative	17 (71%)	5 (71%)	12 (71%)	
Positive	1 (4%)	1 (14%)	0 (0%)	
Unknown	6 (25%)	1 (14%)	5 (29%)	

^⊥^ Only patients with invasive cancer were checked for HER2neu expression. ** HER2-negative includes: HER2 0, 1, 2+ (FISH-negative).

## Data Availability

Data are available upon request and approval from the corresponding author.

## Supplementary Materials

The supporting information can be downloaded at: https://www.mdpi.com/article/10.3390/ijms25179587/s1.
