# Supplementary material for: The Effect of Statins on Markers of Breast Cancer Proliferation and Apoptosis in Women with In Situ or Early-Stage Invasive Breast Cancer
Source: Int J Mol Sci. 2024 Sep 4;25(17):9587. doi: 10.3390/ijms25179587 (PMC11395452; doi:10.3390/ijms25179587)
Supplement: Supplementary file 1 [file ijms-25-09587-s001.zip › ijms-3087094-supplementary.pdf]

Table S1. Baseline tumor characteristics of the 28 women enrolled in the study

ID	AGE_ON_STUDY	RACE	Total.Doses	Stage	ER	PR	HER2	Evaluable	Comments
1	47	Black or African American	4	I	Positive	Positive	Negative	0	Received less than 80% doses; Discontinued due to adverse events
2	48	Black or African American	14	I	Negative	Negative	Negative	1	NA
3	55	Black or African American	0	I	Positive	Positive	Negative	0	Did not receive statins
4	42	White	0	II	Positive	Positive	Negative	0	Did not receive statins
5	44	White	0	I	Positive	Positive	Negative	0	Did not receive statins
6	61	Black or African American	0	II	Positive	Positive	Positive	0	Did not receive statins
7	49	White	14	DCIS (0)	Negative	Negative	Unknown	1	NA
8	72	White	16	I	Positive	Positive	Negative	1	NA
9	59	White	12	DCIS (0)	Positive	Negative	Unknown	1	NA
11	73	White	15	I	Positive	Positive	Negative	1	NA
12	69	White	22	DCIS (0)	Positive	Positive	Unknown	1	NA
13	67	White	17	I	Positive	Positive	Negative	1	NA
14	61	White	0	II	Positive	Positive	Negative	0	Patient taken off study due to surgery being cancelled (COVID-19); patient did not received statins
15	63	White	14	DCIS (0)	Positive	Negative	Unknown	1	NA
16	63	White	14	DCIS (0)	Positive	Positive	Unknown	1	NA
17	73	White	14	I	Positive	Negative	Negative	1	NA
18	71	White	0	DCIS (0)	Negative	Negative	Unknown	0	Patient withdrew
19	67	White	14	I	Positive	Positive	Negative	1	NA
20	70	White	14	I	Positive	Positive	Negative	1	NA
21	64	White	14	I	Positive	Positive	Negative	1	NA
24	59	White	14	DCIS (0)	Positive	Positive	Negative	1	NA
26	49	White	13	I	Positive	Positive	Negative	1	NA
27	57	White	14	I	Positive	Positive	Negative	1	NA
28	60	Black or African American	27	I	Positive	Negative	Negative	1	NA
